# What influences a person’s willingness to share health information for both direct care and uses beyond direct care? Findings from a focus group study in Ireland

**DOI:** 10.12688/hrbopenres.13548.1

**Published:** 2022-05-10

**Authors:** Sarah Jane Flaherty, Catherine Duggan, Laura O'Connor, Barbara Foley, Rachel Flynn

**Affiliations:** 1 1. Health Information and Quality Authority, Cork, T12 Y2XT, Ireland

**Keywords:** health information; digital health; healthcare; public engagement; eHealth; consent model

## Abstract

**Background: **The sharing of health information is invaluable for direct care provision and reasons beyond direct care, such as for health services management. Previous studies have shown that willingness to share health information is influenced by an individual’s trust in a healthcare professional or organisation, privacy and security concerns, and fear of discrimination based on sensitive information. The importance of engaging the public in policy and practice development relating to the use and sharing of health information has been identified as an essential step for countries to take. This study’s aim was to examine the factors that influence the Irish public’s willingness to share their health information as part of a national public engagement on health information.

**Methods:** A qualitative study using online focus groups was conducted as part of a wider national public engagement on health information. Participants were purposively recruited from a combination of public, patient, and service user groups in Ireland. Focus group interviews were audio-recorded, transcribed verbatim and analysed using inductive content analysis.

**Results:** In total, 85 participants took part in 14 focus groups between January and March 2021. Two major themes were identified, trust and personal and public benefits of sharing health information. The ability to exercise control over personal information, perceived transparency of the process, and the extent to which the healthcare service was viewed as confidential, all influenced the level of trust a person held. Perceived benefits were influenced by the extent to which participants believed information sharing would support improved care or provide broader public benefit, and balanced against the potential for personal harm.

**Conclusions:** The findings allow for new insights into the views of the public on the use and sharing of personal health information and can be used to inform the development of a consent model for health information.

## Introduction

Health information is primarily used to inform direct patient care and is often shared between health and social care professionals to inform care provision. It can also be used for purposes beyond direct care, such as to inform health service management and planning; to identify how care may be improved; to support health research; and to inform policy development ^1^.

Many people are willing to share their health information for purposes beyond direct care as they believe that it contributes to the ‘common good’ ^2^. However, current evidence illustrates that willingness to share is influenced by an individual’s trust in the healthcare professional or organisation, which is based on trust in their competence and motivation ^2– 4^. A systematic review of attitudes towards the use of health information for research reported a greater willingness to share if information is de-identified, although consent was still important for many and essential for building trust ^5^. This review also reported that concerns in relation to the privacy and security of health information influenced willingness to share, particularly for perceived sensitive information, such as information on sexual health or drug use. A systematic review of public views on the use of health information for research also reported a fear of discrimination from government agencies and insurance companies if sensitive information was shared ^2^.

In Ireland, there are plans for greater introduction of eHealth initiatives ^6^. These are likely to increase the availability and utility of health information ^3, 7^. Evidence from other countries suggests that public engagement is necessary in advance of such a transition to understand the preferred approach in terms of sharing health information and addressing public concerns ^8, 9^. Consequently, it is important to capture the Irish public’s perspective to adequately inform policy and practice. Furthermore, much of the current literature ^2, 4, 5, 10, 11^ focuses on the sharing of information for research purposes. This may not represent opinion towards the sharing of information for other purposes. It is important that such a gap is addressed to ensure policy and practice is relevant to the diverse uses of health information.

In Ireland, a national public engagement was conducted with the aim of understanding the public’s views on the collection, use and sharing of health information. In the first stage, a nationally representative survey was conducted ^12^. It found that most people view the sharing of health information as important and generally have high trust in healthcare professionals to keep information secure. Sharing sensitive information and the use of health information for commercial reasons were concerns for some. Greater clarity on the information sharing process, removal of identifiable information, and greater access to and control of personal information were identified as ways to improve public acceptance of health information sharing.

In the second stage of the national public engagement, focus groups were conducted to gain a deeper understanding of the public’s views on the collection, use and sharing of health information. The specific objectives of the focus groups were to:

1.   examine the factors that influence trust in health and social care professionals and organisations;

2.   explore concerns in relation to the collection, use and sharing of health information, and opportunities to address such concerns; and

3.   explore views and experiences in relation to the use of electronic health records.

The aim of this paper was to examine the factors that influence an individual’s willingness to share their health information, in relation to the provision of direct care and purposes beyond their direct care, using the focus groups conducted as part of the national public engagement in Ireland.

## Methods

A descriptive qualitative design was employed with focus groups chosen as the method for data collection. Focus groups were held online due to coronavirus disease 2019 (COVID-19) public health restrictions.

### Ethical approval

Ethical approval was obtained for this study from the Royal College of Physicians of Ireland Research Ethics Committee (Reference Number: RCPI RECSAF 130). Approval was granted on 30 September 2020.

### Participants and recruitment

Participants were purposively recruited from a combination of members of the public, patient representatives, and people using particular services. It was planned that one focus group would be facilitated with each of the groups of people that used particular services, two focus groups would be held with patient representatives and three groups would be held with members of the public. It was anticipated that, in line with recommended practice, an average of six to eight participants would be recruited for each focus group ^13^. For the focus groups with members of the public, participants were recruited from an existing panel of people that is managed by an external market research agency, who had previously consented to being contacted to partake in research studies. All participants were invited, via email, to take part in a focus group on the collection, use and sharing of health information. Interested individuals were asked to complete an online questionnaire, using the Askia platform, which assessed their trust and comfort level in relation to the sharing of health information in different scenarios and were subsequently categorised into one of three groups based on their responses. There was a group that was positive towards health information sharing, one that was neutral towards information sharing, and one that had negative attitudes towards the sharing of health information.

For patient representative groups and people using particular services, relevant representative organisations from a range of locations across Ireland were contacted, via email, with information on the study and expected commitment for participants. Additional information, via email or phone, was provided upon request and any queries were addressed. Representative organisations subsequently shared this information with their network. Individuals were asked to inform the representative organisation if they were interested in participating. Contact details for these individuals were subsequently shared with the research team via email.

For all focus groups, interested individuals were sent, via email, an information leaflet, and information specific to their focus group, including broad topics to be discussed. Signed informed consent was collected from all participants prior to their participation. For groups with patients and people that used particular services, documents were tailored for each group based on feedback from each relevant representative organisation. Participants were asked if additional supports were required before, during, and after the focus group and supports were arranged where needed.

### Data collection

All focus groups took place online using an online meeting platform (Zoom ^14^) between January and March 2021. Participants were sent login details in advance and invited to participate in a test call before the focus group to ensure access was possible. The majority of participants connected to the online focus group from their homes. Some participants were located in the offices of the representative organisation (people using addiction services), residential settings (people using disability services), or temporary accommodation (people using addiction services and people using homeless services). A facilitator, moderator, and note-taker were present at all focus groups. Public focus groups were facilitated by a research team from an external market research agency (Behaviour & Attitudes) and the remaining focus groups were facilitated and moderated by researchers from the lead organisation (BF, CD, and SJF).

In all focus groups, an initial introduction was provided which introduced members of the research team, and included an overview of ground rules for the focus group, and a brief presentation on health information, project background and key findings from the national survey. A topic guide was developed in line with study objectives (see extended data). The standard topic guide was used for the public focus groups. For patient groups and people that used particular services, the guide was tailored to the needs of each group based on input from each representative organisation. After each discussion, a summary of the key discussion points was presented by the moderator to get validation from participants that it reflected their opinions and was an accurate representation of the discussion.

Focus groups lasted between 60 – 90 minutes. All focus groups were audio recorded and transcribed verbatim, and subsequently anonymised. Notes recorded by the note-taker were written up, and no personal details were recorded. Focus groups were concluded when the research team agreed that there was sufficient representation from across the range of targeted stakeholder groups. Transcripts and field notes were imported to the qualitative data analysis software NVivo 12, to facilitate data organisation, management, and analysis. 

### Data analysis

Inductive content analysis was undertaken and the Framework Method was employed as the analytical method ^15^. Two researchers (CD & SJF) undertook data analysis. As soon as possible after the focus groups, they each read each transcript and associated notes to familiarise themselves with the discussions. A process of open coding was undertaken by CD on the initial seven focus groups, where codes related to substantive points of interest were identified. Codes were grouped together into categories to form an initial analytical framework and discussed with SJF to ensure agreement on their application to the data and gain a clear understanding of the framework for subsequent coding. The initial coding framework was then applied to all transcripts. Developing the coding framework was an ongoing process undertaken by both CD and SJF, where codes were adapted, added, or removed based on additional readings of the transcripts. Each transcript was initially coded by one of the researchers and subsequently coded by the other researcher to ensure the framework was applied appropriately and consistently. SJF undertook initial theme development where similarities and differences between codes were identified and grouped to generate initial themes. A narrative summary of each theme was developed which was underpinned with relevant quotes. Initial themes were subsequently discussed with CD and updated to reflect this discussion while ensuring they continued to accurately reflect data. Once initial themes were agreed, a summary of themes was discussed with the wider research team, and adapted in line with feedback. Relevant literature was consulted to further refine and contextualise themes with ongoing discussion between members of the research team to ensure credibility.

### Reflexivity

All focus group facilitators were female researchers. Facilitators of the focus groups with members of the public work for a private research company and have considerable experience in facilitating focus groups in the area of social research. Facilitators of the remaining focus groups work for a public sector organisation and all have qualifications in the area of health, work in the area of health information and are experienced and trained in conducting focus groups with different population groups. Introductions and presentations at the beginning of each focus group facilitated the building of a relationship between researchers and participants and ensured participants were aware of the rationale for conducting the focus groups. 

## Results

### Sample

In total, 85 participants took part in 14 focus groups ( Table 1). An additional seven people agreed to participate but withdrew before the focus group took place. 

**Table 1.** Composition of focus groups on attitudes to the collection, use and sharing of health information in Ireland.

	Participant Group	Number of Participants
1	People using addiction services	6
2	People using disability services	6
3	People using homeless services	4
4	People using mental health services	7
5	Representatives of migrant and asylum seeker communities	6
6	Patient representatives (1)	8
7	Patient representatives (2)	7
8	Members of the public (1)	7
9	Members of the public (2)	5
10	Members of the public (3)	6
11	People using sexual health services	4
12	People from the Traveller community	4
13	16 – 17-year-olds (1)	8
14	16 – 17-year-olds (2)	7
**Total**		**85**

### Findings

**Figure 1.**
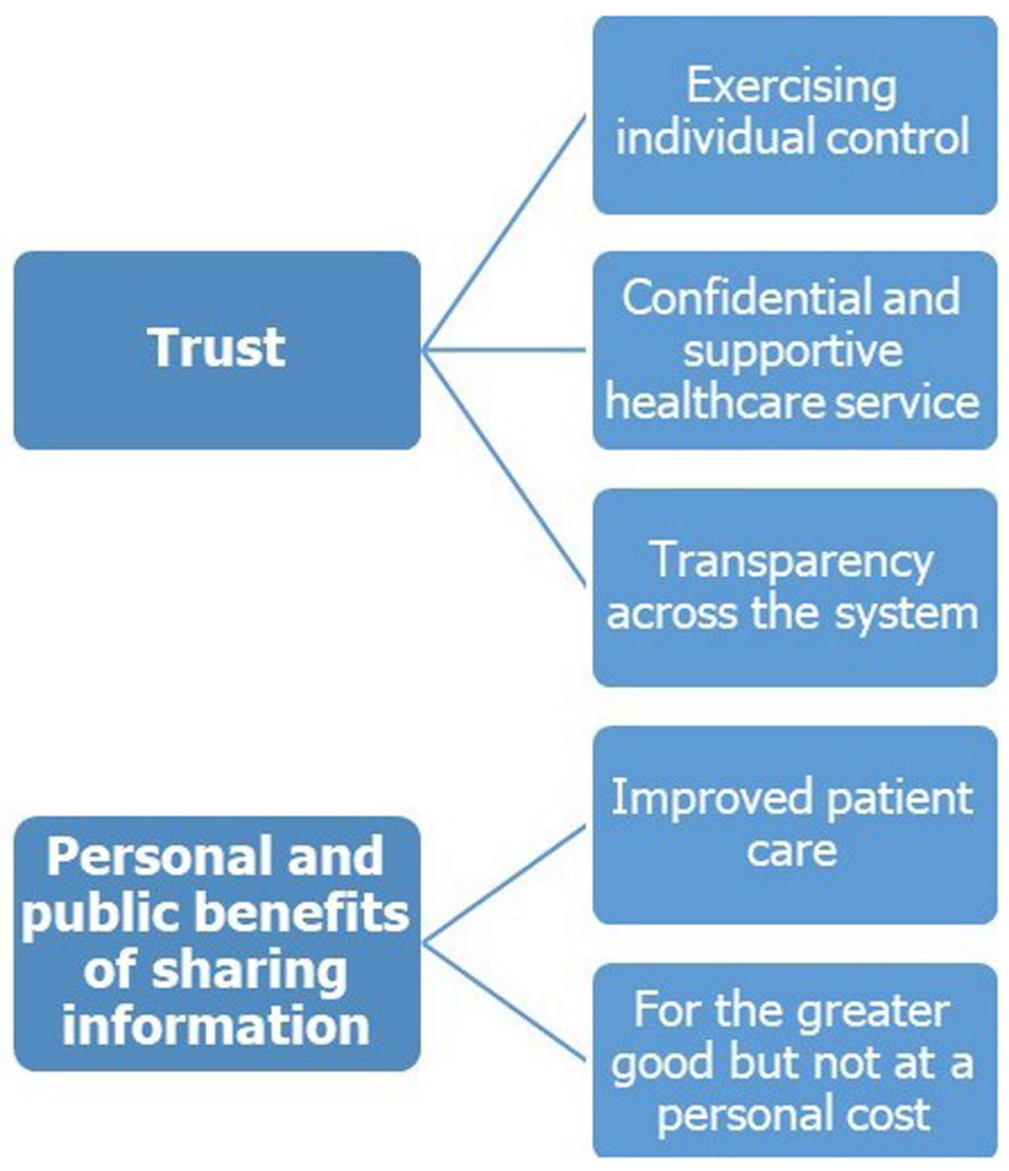
Major themes and sub-themes identified relating to factors that influence willingness to share health information.

Two major themes, underpinned by five sub-themes, were identified ( Figure 1).

### Trust

***Exercising individual control***. There was clear agreement across all groups that people should have greater access to, and control over, their health information as they believe that it belongs to them: “ *it’s your file and you should know what they’re writing…it’s your life and…you should be allowed to look at it” (Person using disability service)*. They viewed such control as an important aspect of trust as it would contribute to feelings of empowerment and allow greater participation in own care. Some participants expressed often feeling powerless in their interactions with the health service, and perceived a lack of open communication from healthcare professionals: “ *you don’t know what’s been said about you…they just write it down and that’s the end of it, you’re not participating in your care…I just find that very frustrating” (Person using mental health service).* These participants felt that greater control would help to address this imbalance and build trust in healthcare professionals. Many participants expressed that greater control was important so that they could review the accuracy of their health information. For people representing the migrant and asylum seeker communities, language barriers and inadequate translation services sometimes led to inaccurate information being recorded: “ *if I don’t necessarily understand English, speaking to my GP I probably do not explain to him exactly what it is I want to pass on” (Representative of migrant and asylum seeker community)*. The majority of participants believed that being able to control personal information would increase trust in the health information system and the different ways in which this information is used.

Some participants, however, expressed the need for caution. Some participants from the patient representative groups believed that it may not be appropriate for people with mental health issues to have full access to their own information as it may have a negative impact on their mental health: *“I don’t think a lot of mental health patients need some of the information…it can be detrimental to their recovery…I would be worried about access to your information (Patient Representative)”.* There were also concerns about people accessing information in the absence of a healthcare professional explaining results, such as when dealing with a terminal diagnosis. Others were concerned about people being able to remove important information. While many participants agreed that there should be greater personal control, there was consensus that safeguards need to be in place around accessing or editing certain types of information and that supports are needed to help people review and interpret their information: “ *there are certain illnesses will need a degree of oversight and…[there] needs to be supports in place to help people even…with the terminology” (Patient Representative)*.

There was general agreement across all groups that health information must be accessible to all regardless of literacy level (health or digital literacy); presence of a disability; spoken language; and resources available, such as mobile or computer access. Participants emphasised that careful consideration is needed on the requirements of different groups to ensure accessibility: “ *it needs to be easy for people with disabilities…easy passwords for them to understand…if it’s complicated… they’ll get distressed” (Person using disability service).* For many, the medical terminology used was viewed as confusing and they emphasised the need for change to allow greater involvement in own care: “ *it is the way they are written…in the doctor language*… *people who have below average [literacy] levels don’t understand what’s being written about them” (Patient Representative).*

***Transparency across the system***. For most participants, there was a lack of understanding of the information sharing process. This contributed to mistrust in the process which negatively influenced a person’s willingness to share. Participants wanted to be more clearly informed of the process: “ *if you are sharing somebody’s information they [need to be] fully aware of where it’s going, why it’s going there, what it’s being used for” (Patient Representative).* Some participants emphasised the need to ensure that any resources explaining how their information is collected, used and shared must be accessible and *“something we could relate to…something we could understand*” *(16 – 17-year-old)*. It was expressed that providing written information alone was not adequate and healthcare professionals were responsible for ensuring people had sufficient understanding.

A small number of participants across all focus groups spoke about the importance of being asked for their consent for the use of their information, especially for purposes beyond their direct care. This appeared to be mostly focused on the sharing of sensitive information as these participants fear discrimination either in their engagement with the health service or elsewhere. For example, asylum seekers and undocumented migrants fear that information will be shared with immigration or other government authorities: *“there’s an instant fear…you are going to come to the attention of the guards [police] or Immigration…it has to be very very clear…what information is shared” (Representative of migrant and asylum seeker community).* These participants felt that improved transparency of the information sharing process would alleviate some of these concerns, and increase their willingness to share information.

The ability to view who had accessed personal health records was considered by many participants as a means of increasing transparency. They viewed it as a deterrent to inappropriate access, such as neighbours or family members accessing personal information for reasons other than direct care: *“[if there are] doctors in the family but they don’t want to deal with them…what’s to stop [the doctors] from looking up their records” (Member of the public)*. Some participants felt that there should be regular audits to address inappropriate access and ensure data breaches were dealt with in a transparent manner. They believed that such approaches would contribute to a more open and transparent system and subsequently contribute to higher levels of trust and willingness to share.

***Confidential and supportive healthcare service***. Many participants trusted healthcare professionals and spoke of relationships built up over time with healthcare professionals, depending on their level of interaction with healthcare services. There was, however, a minority of participants that spoke about their negative experiences which had led to a distrust in healthcare professionals and the health service as a whole. Incidents included not being listened to by healthcare professionals; not treated with dignity and respect; breaches of confidentiality; use of inappropriate language; miscommunication; and being discriminated against based on condition, addiction issues, or medical card ownership. Such negative experiences were more commonly expressed by migrants and asylum seekers and participants with experience of using mental health or addiction services, but negative incidents were mentioned in all focus groups: “ *I do not trust many mental health services anymore, it takes a lot for me to trust even a GP” (Person using mental health services)*.

There was also much discussion on the need to improve confidentiality across the health service. Participants spoke about healthcare professionals openly discussing patient’s details in the corridors, leaving personal files unattended, and delivering diagnoses or requesting personal information in shared spaces. The inadvertent sharing or accessing of sensitive information was of particular importance. Developing an electronic system for the collection, use and sharing of health information was viewed as more private in comparison to paper files: “ *I would agree with the electronic files…because sometimes the [paper] files they walk away from the desk” (Person from the Traveller community).* There were concerns about data breaches and it was emphasised that a system must be secure and properly maintained in all settings to avoid this. This was especially of concern for sensitive information: *“[mental health data] should be double encrypted…it should be highly protected” (Person using mental health services)*.

A common discussion across all focus groups was the need for all health service staff to adhere to relevant rules and procedures in order to embed a culture of trust in the health service. A focus on staff education was considered essential. Participants emphasised that it must include all staff working in the health service with which people have direct contact and not just healthcare professionals: *“[it’s important] that personnel are actually trained in data protection, from the receptionists to the porter, from the ambulance [staff]…technology won’t protect data” (Member of the public).* Some participants believed that there should be serious implications in place for breaching confidentiality. While this may be in place for healthcare professionals, the same deterrents were not perceived to be in place for other health service staff: “ *if you breach [GDPR] you could get into serious trouble…health professionals, you might lose your job…I don’t think it’s as guided and as guarded within the admin side… in your contract it should be stated to respect GDPR…and do training with the staff about the implications of breaching GDPR” (Member of the public).* Participants emphasised that confidentiality was key and must underpin all actions within the health service to build trust.

### Personal and public benefits of sharing information

***Improved patient care***. Participants were generally more willing to share information if they felt that it would improve the quality of their care. They considered it essential that healthcare professionals had access to relevant information to inform direct care: “ *healthcare professionals should have as much information about us as possible because otherwise how can they make a properly informed decision on how to take our healthcare forward” (Patient Representative).* One participant highlighted that such access was crucial for those who are not able to communicate verbally: “ *[my daughter is] non-verbal so it’s extremely important that information flows around her completely and that everybody gets to know” (Representative of person using disability services).* Participants believed that improved access for healthcare professionals would reduce the need for individuals to repeat information or speak about distressing elements of their medical history, such as addiction or a miscarriage: “ *I see multiple specialists over four different hospitals in three different counties so I need them all to be able to look at what the other one has written…[otherwise] I have to go around telling everybody everything…and a lot of patients can’t do that” (Patient Representative).* Many participants also believed that greater access to information was more efficient as it would reduce the time that healthcare professionals spent gathering information, subsequently allowing more time for direct care.

Within all focus groups, there were differing views on whether healthcare professionals should have access to all of an individual’s health information. Some participants were concerned about sharing sensitive information, such as mental health or sexual health information, as they did not consider it relevant in certain instances. They believed that only information directly relevant to the care episode should be available. Many of these concerns arose from a fear of discrimination in the care received: “ *I would be very worried about having a physical illness and how I’d be treated…when they see I’ve got a psychiatric diagnosis …I’ve heard stories about people having a really rough time because they’ve got a psychiatric diagnosis” (Person using mental health services).*

Other participants felt that only a healthcare professional could decide on the relevancy of information: “ *I would not consider myself competent to decide whether that is relevant or not …what we might think is not relevant could end up being relevant…we just don’t realise it at the time” (Patient Representative).* Some participants believed that a lack of information in some circumstances may be detrimental: “ *[the doctor] might not know you’re taking [medication]…the amount of drugs which you’re taking could cause a problem or maybe an overdose…it’s good for a doctor to know” (Person using addiction services).* Participants in the 16 - 17-year-old group discussed the potential broader impact of separating mental health information from physical health information. They felt that *“if we separated mental and physical health, it would add to the stigma around mental health”,* and argued that *“[mental health issues] should be normalised and seen to be on the same level as breaking your leg”.* There was general agreement within groups that certain healthcare professionals, such as an individual’s GP or emergency medicine doctors, should have access to all information as it may be important in a crisis, and that essential information, such as name, age, blood type, allergies, or disability, could be made accessible to all healthcare professionals.

Greater implementation of electronic health records was generally welcomed by participants as they were viewed as a means of allowing easier access and sharing of information for healthcare professionals. There was general agreement across groups that electronic records would ensure an up-to-date and comprehensive account of an individual’s health that would contribute to more timely and appropriate care: “ *I would have been delayed in the clinic while they were waiting to find my chart [from] when I was in another clinic either that morning or yesterday…so I think electronic is the way to go” (Person using sexual health services)*. Some participants were more cautious about their introduction and commented that consideration needs to be given to people with low digital literacy, and the impact of IT failures and inadequate IT infrastructure on direct care.

***For the greater good but not at a personal cost***. There was a general willingness among all participants to share information for purposes beyond direct care if it provided personal or public benefit, such as improvement in quality of care: *“it’s important the information is shared …they can experiment with different things to find out what works and what doesn’t work” (Person using disability services)*. Concerns emerged, however, in relation to the sharing of information outside of the public sector as participants questioned the motives of private organisations: *“my only concern would be that pharmaceutical companies…get access to people’s data because their motives may not be as pure as…government agencies” (Member of the public)*. However, participants were typically willing to share information if they viewed the organisational motive as being in the public interest. For example, many participants viewed sharing information with pharmaceutical companies positively as they believed that these companies conduct important research, but they were less willing to share their information if it was being used for financial gain with limited public benefit: *“I think that the greater good is definitely served by sharing a significant element of information…I don’t think we should be giving information for cosmetic[s]” (Patient Representative).*

Some participants expressed concern that information would be shared for non-healthcare purposes, such as with immigration agencies, insurance companies, and prospective employers. People feared discrimination due to existing or previous circumstances or health conditions, and believed that information should not be shared if it was going to have a detrimental personal impact. For example, all participants in the focus group of people that used addiction services felt that there was a stigma attached to addiction and some commented that this stigma persisted long after addiction ceased: *“[I’m] 23 years away from addiction…living a completely different life but when you go to apply for…health insurance…the medical reports might show stuff that you don’t really want to mention…then they might say you’re not entitled to…medical cover” (Person using addiction services)*.

Sharing anonymised information was viewed by most participants as more acceptable for purposes beyond direct care: “ *all of the information should be de-identified so there shouldn’t be anything that would link back to an individual” (Patient Representative).* For some participants, anonymisation addressed their concerns in relation to sharing information with private organisations as it offered individual privacy, although concerns relating to organisational motives remained for others. Some participants highlighted that anonymisation was not possible for smaller communities, such as homeless service users or minority ethnic groups: “ *sometimes in small places [anonymisation] can be very difficult to do because the homeless population itself is quite small and the presentations…[are] quite unusual [so] it’s very hard to anonymise” (Representative of homeless services)*.

## Discussion

The present research explored people’s views on the collection, use and sharing of health information for both direct patient care and purposes beyond direct care in Ireland. Trust and the perceived personal and public benefits of sharing health information were identified as major influences on a person’s willingness to share their information.

The finding that trust was an important factor aligns with existing literature which highlights trust as a key influence on willingness to share ^10, 16^. Trust is a complex concept and can be influenced by personal characteristics and organisational, structural and contextual factors ^2, 17^. In the present research, multiple factors influenced the level of trust a participant held which is similar to findings presented elsewhere ^2, 4, 18^ and re-emphasises that trust is situational and shaped by different interactions of multiple factors ^3^. ‘Understanding Patient Data’, a UK-based organisation focused on bringing the views of patients and the public to policymakers, argues that emphasis should be placed on the trustworthiness of an organisation rather than on individual levels of trust ^17^. Trustworthiness focuses on practical and objective actions that can be implemented by the organisation ^17^. In an essay proposing a model of trustworthiness, the dependability, competence and responsiveness of an organisation are key elements of trustworthiness and need to be clearly demonstrated to be viewed as trustworthy ^19^.

Drawing on the findings of this study and evidence from elsewhere, there are a number of practical actions that could be taken to demonstrate trustworthiness. Increased transparency of the information sharing process and providing greater control to individuals have been identified as important potential actions, both in this research and in a range of different studies ^2, 18, 20, 21^. In examining the patient experience of electronic records implementation, greater patient control contributed to a feeling of empowerment and greater involvement in own care, which subsequently supported trust building ^21, 22^. Evidence suggests that such control and transparency is important for all patients with Holm *et al.* ^18^ finding no differences in their importance based on whether someone was a frequent user of health services or not. It is important to remember, however, the multiple factors that influence trust, and various complementary strategies are needed depending on the specific context ^2, 3^.

In this study, participants were typically more willing to share information if there were perceived benefits for either individual care or for the broader population, such as the development of new treatments and technologies. Similar findings were reported in a systematic review into public views on the use of data for research, where some people spoke of a ‘social responsibility’ to share information for public benefit ^2^. In the current study, such benefits were evaluated against the risk of sensitive information being shared inappropriately and its potential consequences, such as discrimination in care or in interactions with immigration agencies and insurance companies. Similar concerns are voiced elsewhere in the literature ^11, 23– 25^. Much of this literature considers sexual health and mental health-related information as sensitive. Findings from the wider national public engagement survey undertaken in Ireland ^12^ illustrate that many types of information can be considered sensitive, including pregnancy and reproductive information, addiction issues, and sexual orientation. This suggests that a broader perspective of ‘sensitive’ information should be considered in future research and practice. It will also be valuable to examine if there are differences on willingness to share depending on what information is considered sensitive.

One of the key discussion points in the focus groups was the willingness to share information with public or private organisations. While findings from other studies report that people generally don’t approve of sharing information with private organisations ^2, 26^, participants in this study expressed a more nuanced view. Information sharing was typically more acceptable if the organisation’s motives were viewed as being in the public interest. Participants in workshops undertaken with public and patient representatives in the United Kingdom expressed similar views ^27^. They considered sharing information with private organisations important if it provided public benefit, but stated that control of information must be retained by the public. In the current focus groups, many participants were more comfortable with non-identifiable information being shared. This is in line with the findings of a systematic review of health consumer attitudes, where some viewed the collective benefit of information sharing as overriding any right to individual privacy ^5^. There were, however, still some participants in the current focus groups who were reluctant to relinquish such control illustrating the complexity of perspectives. 

As eHealth initiatives advance and bring change to health information systems, these findings can be used to inform relevant policy and practice and ensure that they meet the needs of all health and social care service users. In 2021, the implementation of a new GP data collection framework was paused in England due to a lack of appropriate engagement and concerns about consent procedures ^28, 29^, highlighting the importance of adequate public engagement. The findings of this study show that certain factors are considered relevant to both the sharing of information for direct care and purposes beyond direct care. Consequently, the factors identified in this study should be considered at all stages of the information sharing process and integrated into policy and practice, where relevant.

### Strengths and limitations

Much of the current literature focuses on the sharing of health information for research. A key strength of this study is that it explored opinions on multiple uses of health information. Another important strength is the purposive inclusion of population groups that may have specific needs in relation to health information, such as migrant groups or users of sexual health services, which has been highlighted as a gap ^3^. Furthermore, we spoke directly to people from these groups rather than solely to their representatives. This approach is noteworthy as it provides a direct voice to individuals rather than a potential interpretation of their views and experiences.

Due to the involvement of marginalised communities, participants’ sociodemographic data were not collected as it may be considered sensitive by some. We also wanted to reduce the potential burden of participation to encourage involvement. This does not allow us to examine differences by traditional demographic categories, such as age and gender, and limits the ability to make comparisons with particular research. However, we do not think that this limitation undermines the current findings as sufficient context was captured in each focus group to inform the analysis.

Due to the public health restrictions in place due to COVID-19, all focus groups were held online. This may have increased the potential for participation for certain groups, such as those with mobility issues or lack of accessible transport. It may, however, have limited the involvement of other groups, such as those with limited digital literacy or poor internet access. We worked with representative organisations to address these concerns and support was provided where needed. It is possible that some individuals did not participate due to it being online which may have resulted in an understatement of the influence of digital literacy and access on an individual’s willingness to share health information. 

## Conclusion

This study explored the factors that influenced people’s willingness to share health information for both direct care and purposes beyond direct care in Ireland. The findings highlight the importance of building public trust and organisational trustworthiness, and promoting awareness of the personal and public benefits of sharing health information. Such insight can be used to inform the development of consent models and ensure that they meet the needs of the public and health and social care professionals. 

## Data availability

### Underlying data

Focus group transcripts used in this analysis cannot be made accessible as the participants include those from vulnerable groups and contain sensitive information. Ethical approval and participant consent restricts access to the raw data to the research team. Select quotes may be made available on request from the corresponding author ( sjflaherty@hiqa.ie).

### Extended data

Open Science Framework: What influences a person’s willingness to share health information for both direct care and uses beyond direct care?: findings from a focus group study in Ireland. https://doi.org/10.17605/OSF.IO/FXEPU ^30^

This project contains the following extended data:

- Topic guide
- Coding framework
- COREQ Checklist.

Data are available under the terms of the Creative Commons Attribution 4.0 International license (CC-BY 4.0).
