# Supplementary material for: What influences a person’s willingness to share health information for both direct care and uses beyond direct care? Findings from a focus group study in Ireland
Source: HRB Open Res. 2022 Oct 24;5:36. Originally published 2022 May 10. [Version 2] doi: 10.12688/hrbopenres.13548.2 (PMC9411973; doi:10.12688/hrbopenres.13548.2)
Supplement: Peer review history

# Peer review: Reviewer response for version 1

**DOI:** 10.21956/hrbopenres.14790.r32672

**Published:** 2022-08-30

**Authors:** Neil O'Hare

**Affiliations:** 1 School of Public Health, Physiotherapy and Sports Science, University College Dublin, Dublin, Ireland

**General Comments:**

 The reviewer found this an interesting article and quite pertinent to work on-going in Ireland around data capture electronically and subsequent sharing of same to support the necessary interests of patient care, audit and research. The article was well written with clear goals, well referenced, and findings presented. There are some specific comments below but these should not detract from the overall thrust of the paper.

1. Abstract – Results (and in the overall findings): It would be interesting to see the results pre and post C-19 pandemic. The engagement with the public around the sharing of health information certainly changed during Covid in a more positive and open way (or at least appeared to be the case) and it will be interesting to see if this reverts to pre-Covid feelings in time. This does not call in to question the findings, rather potentially time-bounds them.
2. Methods – paragraph before the start of the “Data Collection” section. “For groups with patients and people that used particular services…..”. A definition or examples of “particular services” would be beneficial. In a way, most patients use particular services, but I suspect the authors may mean disability services or mental health services, etc.
3. Table 1: 40% of the Participant Groups were not related to a particular service or grouping (i.e. were general users). Does this reflect the health service user distribution or could the results be interpreted as being skewed away from the “general” users?
4. In the paragraph immediately before “Transparency across the system”, the authors talk about medical terminology being confusing. This raises an interesting point – is the medical chart a record for the patient or for the staff treating the patient to aid in information sharing and delivery of care. If this is the case, as it can be strongly argued that it is, then the medical terminology should be user appropriate and may therefore not be regarded as confusing.

Is the work clearly and accurately presented and does it cite the current literature?

Yes

If applicable, is the statistical analysis and its interpretation appropriate?

Not applicable

Are all the source data underlying the results available to ensure full reproducibility?

Yes

Is the study design appropriate and is the work technically sound?

Yes

Are the conclusions drawn adequately supported by the results?

Yes

Are sufficient details of methods and analysis provided to allow replication by others?

Yes

Reviewer Expertise:

Healthcare Informatics

I confirm that I have read this submission and believe that I have an appropriate level of expertise to confirm that it is of an acceptable scientific standard.

# Peer review: Reviewer response for version 1

**DOI:** 10.21956/hrbopenres.14790.r32676

**Published:** 2022-08-30

**Authors:** Ciara Heavin

**Affiliations:** 1 Department of Business Information Systems, Cork University Business School, University College Cork, Cork, Ireland

The paper investigates the factors that influence the Irish public’s willingness to share their health information. Using a qualitative approach, the researchers report their engagement in a public consultation process to elicit the views of individual citizens in terms of their attitude towards sharing personal health information. A thematic analysis of focus group transcripts was undertaken. The authors investigate an important and timely topic given the EU 2030 target in health information https://www.irishtimes.com/health/2022/08/24/electronic-health-records-state-has-huge-mountain-to-climb/.

 In total, 85 participants took part in 14 focus groups between January and March 2021. We do not get the gender breakdown of participants; this could be useful as we know from existing research that attitudes to health and health information can be gendered. Also, we do not get a sense of the inclusion of representative over 65+, this is a group that may be disproportionately impacted by some of the questions being asked here. 

 Did the participants include any representation from the LGBTQ+ community? This is a population that is significantly underserved in terms of access to right kind of healthcare and related supports in Ireland.

 Does the timing of the study have an impact on the results presented? Have the researchers considered/observed any “covid effect” in the data? 

 The data analysis section is quite comprehensive, perhaps it would benefit from a visual to illustrate the steps and researcher engagement at each phase in the analysis.

 I really like Figure 1, it would have been nice to see a more explicit “chain of evidence” in terms of the data analysis from the raw data to the high level themes and sub-themes.

 In the results section, it is not clear what is meant by a “majority” or” a small number” really means in the context of 85 participants. Did the researchers consider using descriptive stats e.g. frequency counting to represent this?  

 In terms of the sampling strategy and the analysis, there seems to be a mix of individual’s personal views being presented alongside representation from specific populations. It is not clear how the “different levels” of opinion are being treated in the results.

 I really like to theme “For the greater good but not at a personal cost” – I think this captures the dilemma that individuals are faced with in terms of sharing sensitive personal health data. This research emphasizes the need to move away from a “one size fits all” approach to health information sharing. The authors refer to building consent models, it is important to consider what standards in “Privacy by Design” in terms of developing/procuring new platforms for information sharing. The authors propose a number of ways that this may be achieved. This is a complex socio-technical problem that requires significant resources to achieve the EU 2030 target. Great to see this kind of research coming to fruition now.

Is the work clearly and accurately presented and does it cite the current literature?

Yes

If applicable, is the statistical analysis and its interpretation appropriate?

Yes

Are all the source data underlying the results available to ensure full reproducibility?

Yes

Is the study design appropriate and is the work technically sound?

Yes

Are the conclusions drawn adequately supported by the results?

Yes

Are sufficient details of methods and analysis provided to allow replication by others?

Partly

Reviewer Expertise:

Information Systems, Health Information Systems

I confirm that I have read this submission and believe that I have an appropriate level of expertise to confirm that it is of an acceptable scientific standard.

# Peer review: Reviewer response for version 1

**DOI:** 10.21956/hrbopenres.14790.r32673

**Published:** 2022-08-25

**Authors:** Lucy Hederman

**Affiliations:** 1 ADAPT Centre for Digital Content, University of Dublin Trinity College, Dublin, Ireland

This paper provides an interesting addition to knowledge of people's attitudes to sharing of health data. It largely confirms previous studies, but the focus on secondary uses other than research (such as service planning and quality improvement) is new and useful. The research involved a series of focus groups; the diversity of participant groups (e.g. users of a variety of services, patient representatives, general public) is a strength of the research. The effort put into study design and recruitment may have contributed to more nuanced positions than previous studies, such as attitudes to sharing with private entities.

 I am not an expert on thematic analysis. As far as I can tell it is done appropriately. Raw data from the focus groups is not made available for confidentiality reasons. The report is clear and well-written.

 Some suggestions for improving the paper follow.

1. It might be useful to mention near the start what other (non-research) secondary uses were discussed as this is identified as a key strength of this study. I note that service planning and quality improvement are mentioned in the focus group protocol, for example.
2. In the sentence "... the need for all health the need for all health service staff to adhere to relevant rules and procedures in order to embed a culture of trust in the health service", I wonder, should it refer to a culture of trust in *how data is handled* in the health service? Trust in healthcare is mostly about the quality of care, etc. But presumably, these discussions were focused on trust in how data is protected/shared.
3. "A small number of participants across all focus groups ..." in Transparency section - does this really mean that there was a participant in each and every focus group with the reported view? Or that these participants were distributed across all types of participant groups (public, reps, service users)? Or did you just mean that there was a small number of participants?
4. The sentence "This does not allow us to examine differences by traditional demographic categories" would be clearer as "This prevents us from examining ..."

Is the work clearly and accurately presented and does it cite the current literature?

Yes

If applicable, is the statistical analysis and its interpretation appropriate?

Not applicable

Are all the source data underlying the results available to ensure full reproducibility?

Partly

Is the study design appropriate and is the work technically sound?

Yes

Are the conclusions drawn adequately supported by the results?

Yes

Are sufficient details of methods and analysis provided to allow replication by others?

Yes

Reviewer Expertise:

Health informatics

I confirm that I have read this submission and believe that I have an appropriate level of expertise to confirm that it is of an acceptable scientific standard.

# Peer review: Reviewer response for version 1

**DOI:** 10.21956/hrbopenres.14790.r32116

**Published:** 2022-07-26

**Authors:** Sarah Cunningham-Burley

**Affiliations:** 1 Centre for Biomedicine, Self and Society, Usher Institute, University of Edinburgh, Edinburgh, UK

This is a very clearly written paper and succinctly reports on the results of focus group research examining publics’ views on sharing health information for direct care and other uses.  It makes a valuable contribution to our understanding of these issues because of the focus on sharing data for direct care purposes. Much focus of public engagement with respect to data sharing focus on health research.  Similar themes emerge to that identified in prior literature but the paper is nonetheless a worthwhile addition to the evidence available.  There are other strengths to the paper, particularly the care taken to recruit for diversity of experience and also positionality (with respect to the issue under investigation).  This led to some excellent granular data on concerns such as discrimination and stigma.  The authors provide a summary of some prior work, focussing mainly on very recent literature.  This misses some other contributions, albeit about health data for research and I suggest the authors explore this literature on publics and health data research and on public engagement around data a little more extensively.  A few suggestions of relevant literature are given below. This body of already published work reinforces the current paper’s findings about trust, trustworthiness, and control. There have also been other public engagement exercises on these issues, including with respect to use of data during COVID-19.  Finally, the authors might add a little more on the multiple meanings of public engagement and where their work fits (public consultation rather than anything more deliberative), and the relationship between research and engagement.

 A few suggestions regarding literature:

 Aitken, M., Porteous, C., Creamer, E., & Cunningham-Burley, S. (2018). Who benefits and how? Public expectations of public benefits from data-intensive health research. ^1^

 Aitken, M., de St. Jorre, J., Pagliari, C., Jepson, R., & Cunningham-Burley, S. (2016) Public responses to the sharing and linkage of health data for research purposes: a systematic review and thematic synthesis of qualitative studies. ^2^

 Aitken, M., Cunningham-Burley, S., Pagliari, C. (2016) Moving from trust to trustworthiness: Experiences of public engagement in the Scottish Health Informatics Programme.  Science and Public Policy, 1-11. ^3^

Is the work clearly and accurately presented and does it cite the current literature?

Yes

If applicable, is the statistical analysis and its interpretation appropriate?

Not applicable

Are all the source data underlying the results available to ensure full reproducibility?

Partly

Is the study design appropriate and is the work technically sound?

Yes

Are the conclusions drawn adequately supported by the results?

Yes

Are sufficient details of methods and analysis provided to allow replication by others?

Yes

Reviewer Expertise:

Public engagement with health data and research; qualitative health research; medical and family sociology

I confirm that I have read this submission and believe that I have an appropriate level of expertise to confirm that it is of an acceptable scientific standard.

## References

[1] : Who benefits and how? Public expectations of public benefits from data-intensive health research. *Big Data & Society* .2018;5(2) : 10.1177/2053951718816724 10.1177/2053951718816724

[2] : Public responses to the sharing and linkage of health data for research purposes: a systematic review and thematic synthesis of qualitative studies. *BMC Medical Ethics* .2016;17(1) : 10.1186/s12910-016-0153-x 10.1186/s12910-016-0153-x 27832780PMC5103425

[3] : Moving from trust to trustworthiness: Experiences of public engagement in the Scottish Health Informatics Programme. *Sci Public Policy* .43(5) : 10.1093/scipol/scv075 713-723 10.1093/scipol/scv075 28066123PMC5210028
